# Postprandial leucine and insulin responses and toxicological effects of a novel whey protein hydrolysate-based supplement in rats

**DOI:** 10.1186/1550-2783-9-S1-P30

**Published:** 2012-11-19

**Authors:** Ryan G Toedebusch, Thomas E Childs, Shari R Hamilton, Jan R Crowley, Frank W Booth, Michael D Roberts

**Affiliations:** 1 Department of Biomedical Sciences, College of Veterinary Medicine; University of Missouri, Columbia, MO, USA; 2 Research Animal Diagnostics Laboratory (RADIL); Columbia, MO, USA; 3 NIH/NCRR Mass Spectrometry Resource; Washington University, St. Louis, MO, USA; 4 School of Medicine, Division of Endocrinology, Metabolism, Lipid Research; Washington University, St. Louis, MO, USA; 5 Dalton Cardiovascular Research Center; University of Missouri, Columbia, MO, USA; 6 Department of Medical Pharmacology and Physiology, School of Medicine; University of Missouri, Columbia, MO, USA; 7 Department of Nutrition and Exercise Physiology, College of Environmental Health Sciences; University of Missouri, Columbia, MO, USA

## Background

The purpose of this study was: aim 1) compare insulin and leucine serum responses after feeding a novel hydrolyzed whey protein (WPH)-based supplement versus a whey protein isolate (WPI) in rats during the post-absorptive state, and aim 2) to perform toxicological analysis on rats that were fed different doses of the novel WPH-based supplement over a 30-day period.

## Methods

In male Wistar rats (~250 g, n = 40), serum insulin and leucine concentrations were quantified up to 120 min after one human equivalent dose of a WPI or the WPH-based supplement. In a second group of rats (~250 g, n = 20), we examined serum/blood and liver/kidney histopathological markers after 30 days of feeding low (1human equivalent dose), medium (3 doses) and high (6 doses) amounts of the WPH-based supplement.

## Results

In aim 1, leucine levels were significantly higher at 15 min after WPH vs. WPI ingestion (p = 0.04) followed by higher insulin concentrations at 60 min (p = 0.002). In aim 2, liver and kidney histopathology/toxicology markers were not different 30 days after feeding with low, medium, high dose WPH-based supplementation or water only. There were no between-group differences in body fat or lean mass or circulating clinical chemistry markers following the 30-day feeding intervention in aim 2.

## Conclusion

In comparison to WPI, acute ingestion of a novel WPH-based supplement resulted in a higher transient leucine response with a sequential increase in insulin. Furthermore, chronic ingestion of the tested whey protein hydrolysate supplement appears safe.

